# Phagocytosis of Lymphocytes and Erythrocytes by B‐Cell Lymphoma in a Patient With a Clinical Suspicion of Pure Red Cell Aplasia

**DOI:** 10.1002/ccr3.71750

**Published:** 2025-12-29

**Authors:** Tuo Cao, Yuting Li, Xiangming Peng

**Affiliations:** ^1^ Department of Clinical Laboratory, Guangzhou Red Cross Hospital Jinan University Guangzhou China; ^2^ Guangzhou Haizhu District Center for Disease Control and Prevention Guangzhou China

**Keywords:** B‐cell lymphoma, cold agglutinin disease, diagnosis, phagocytosis, pure red cell aplasia

## Abstract

Herein, we report a diagnostically challenging case of a B‐cell lymphoma initially masquerading as pure red cell aplasia (PRCA). The key to diagnosis was the unique finding of phagocytic activity (engulfing erythrocytes and lymphocytes) by atypical mature lymphocytes in both peripheral blood and bone marrow, accompanied by clinical signs of cold agglutination. Despite inconclusive initial morphology for lymphoma and a bone marrow erythroid count that did not fully meet PRCA criteria, flow cytometric immunophenotyping confirmed a clonal B‐cell population. This case underscores that lymphocyte‐mediated phagocytosis is a highly suggestive, albeit rare, indicator of malignancy and can serve as a pivotal clue to uncover an underlying lymphoma obscured by autoimmune phenomena like PRCA.

## Key Clinical Message

The observation of erythrophagocytosis and lymphophagocytosis by mature, atypical lymphocytes in peripheral blood and bone marrow is an exceptionally rare but critical diagnostic clue for underlying B‐cell lymphoma. This phenomenon, especially when coexisting with cold agglutination and erythroid hypoplasia, should prompt immediate investigation for a lymphoproliferative disorder, even in cases initially suggestive of pure red cell aplasia (PRCA).

## 1 Introduction

Pure red cell aplasia (PRCA) is a hematological disorder distinguished by a significant reduction in erythropoiesis, while other cell lineages remain unaffected [1]. PRCA is primarily classified into genetic and acquired forms, with further subdivisions into primary and secondary categories [2]. Acquired PRCA can be further delineated into idiopathic cases and those secondary to a variety of disorders. Secondary PRCA is often linked to various hematological disorders, such as chronic lymphocytic leukemia (CLL) and non‐Hodgkin lymphoma (NHL) [3]. The association between PRCA and lymphoproliferative disorders is well recognized [4]. PRCA may precede the onset of lymphoma, present concurrently with the lymphoid neoplastic disease, or manifest subsequent to the lymphoproliferative disorder [5].

Phagocytosis is a fundamental mechanism for nutrient acquisition in unicellular organisms and is also prevalent across nearly all cell types in multicellular organisms [6]. Professional phagocytes, such as macrophages, neutrophils, monocytes, and dendritic cells, play a crucial role in the removal of microorganisms and the presentation of antigens to lymphocytes, thereby initiating an adaptive immune response [7]. In contrast, non‐professional phagocytes, including fibroblasts and epithelial cells, are incapable of ingesting microorganisms but are essential for the clearance of dead cells and the maintenance of homeostasis [7]. Phagocytosis is characteristic of myeloid‐lineage cells and is exceptionally uncommon for lymphocytes.

In this report, we describe the pivotal role of phagocytosis of lymphocytes and erythrocytes by neoplastic B‐cells—a finding of exceptional rarity—in diagnosing a B‐cell lymphoma that was obscured by a PRCA‐like presentation and cold agglutination. This phenomenon has been rarely reported in the literature. Our case highlights that lymphocyte‐mediated phagocytosis is not merely a morphological curiosity but a highly specific diagnostic indicator of an underlying lymphoproliferative disorder. Increased recognition of this finding is critical, as it should prompt immediate and definitive ancillary testing, such as flow cytometric immunophenotyping.

## 2 Case Presentation

### 2.1 Clinical History

A 70‐year‐old male presented with a 6‐week history of progressive fatigue, dizziness, pallor, and shortness of breath. An initial outside diagnostic workup suggested hypoplastic erythropoiesis, raising the suspicion of pure red cell aplasia (PRCA), which prompted referral for further evaluation.

### 2.2 Diagnostic Assessment

#### 2.2.1 Initial Laboratory Findings and a Diagnostic Dilemma

Laboratory investigations upon admission revealed profound anemia (hemoglobin 34 g/L) with an extremely low reticulocyte count (0.003 × 10^12^/L), consistent with a central failure of erythropoiesis. The mean corpuscular volume (MCV) and mean corpuscular hemoglobin (MCH) were elevated at 109.3 fL and 48.3 pg, respectively. The serum erythropoietin level was markedly elevated (> 750 mIU/mL). These findings initially strongly supported the outside diagnosis of PRCA. However, a critical inconsistency emerged: the bone marrow aspirate showed 9% nucleated erythroid cells, which is above the World Health Organization's diagnostic threshold of < 5% for classical PRCA, thereby challenging the initial diagnosis.

#### 2.2.2 An Unexpected Clue: Cold Agglutination

A pivotal clue was discovered macroscopically: the anticoagulated blood sample formed prominent, sediment‐like aggregates of erythrocytes. This aggregation was promptly reversed upon warming the specimen to 37°C, unequivocally indicating the presence of cold agglutination. Subsequent serological testing confirmed a high‐titer cold agglutinin (≥ 1:512) and a positive direct Coombs test, shifting the diagnostic focus towards an immune‐mediated process complicating the bone marrow failure.

#### 2.2.3 The Pivotal Morphological Discovery: Phagocytic Lymphocytes

Microscopic examination of the peripheral blood and bone marrow aspirate smears then yielded the decisive diagnostic evidence.

**FIGURE 1:**
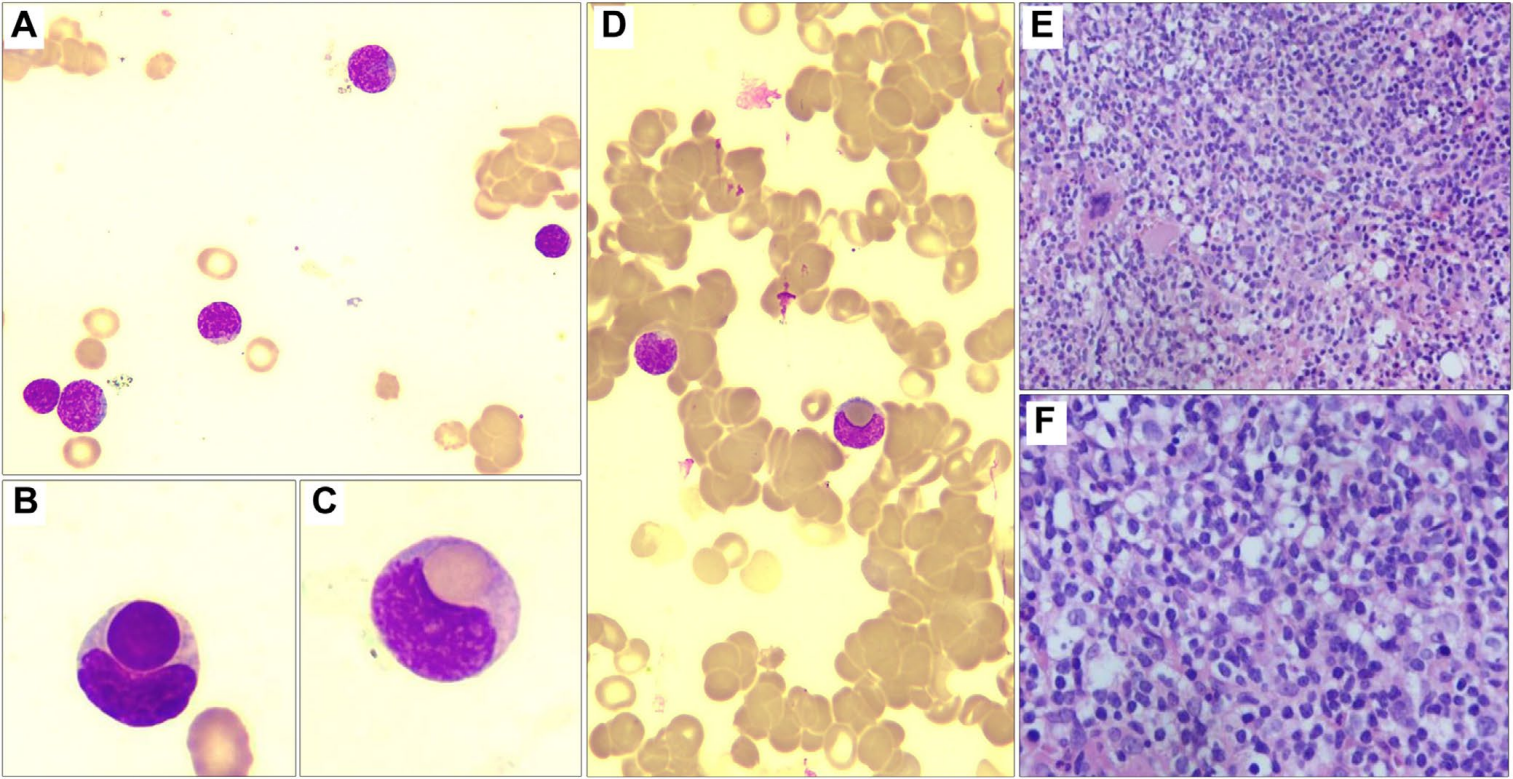
Diagnostic morphological findings in peripheral blood and bone marrow. (A) Peripheral blood smear showing atypical lymphocytes with subtle nuclear irregularities and mild chromatin dispersion. (B, C) Atypical lymphocytes in peripheral blood demonstrating definitive lymphophagocytosis (B) and erythrophagocytosis (C), a key diagnostic finding in this case. (D) Bone marrow aspirate smear showing an atypical lymphocyte engaged in erythrophagocytosis, accompanied by background red blood cell agglutination. (E) Bone marrow core biopsy showing hypercellularity with significant erythroid hypoplasia. (F) Higher‐power view of the bone marrow biopsy revealing an interstitial infiltrate of atypical lymphocytes. Staining and magnification: Panels (A–D) Wright‐Giemsa stain, 100× objective; Panels (E, F) Hematoxylin and Eosin (H&E) stain; 10× (E) and 40× (F) objective, respectively.

1. The peripheral blood smear confirmed marked red blood cell agglutination and revealed a population of atypical lymphocytes with enlarged nuclei and finely dispersed chromatin (Figure 1A,D).
2. Most strikingly, both the peripheral blood and bone marrow smears exhibited unequivocal evidence of phagocytosis: these atypical lymphocytes were actively engulfing intact erythrocytes and other lymphocytes (Figure 1B,C). This phenomenon of erythrophagocytosis and lymphophagocytosis by mature lymphocytes is exceptionally rare and served as a critical indicator of an underlying lymphoproliferative disorder.

### 2.3 Confirmatory Investigations

**FIGURE 2:**
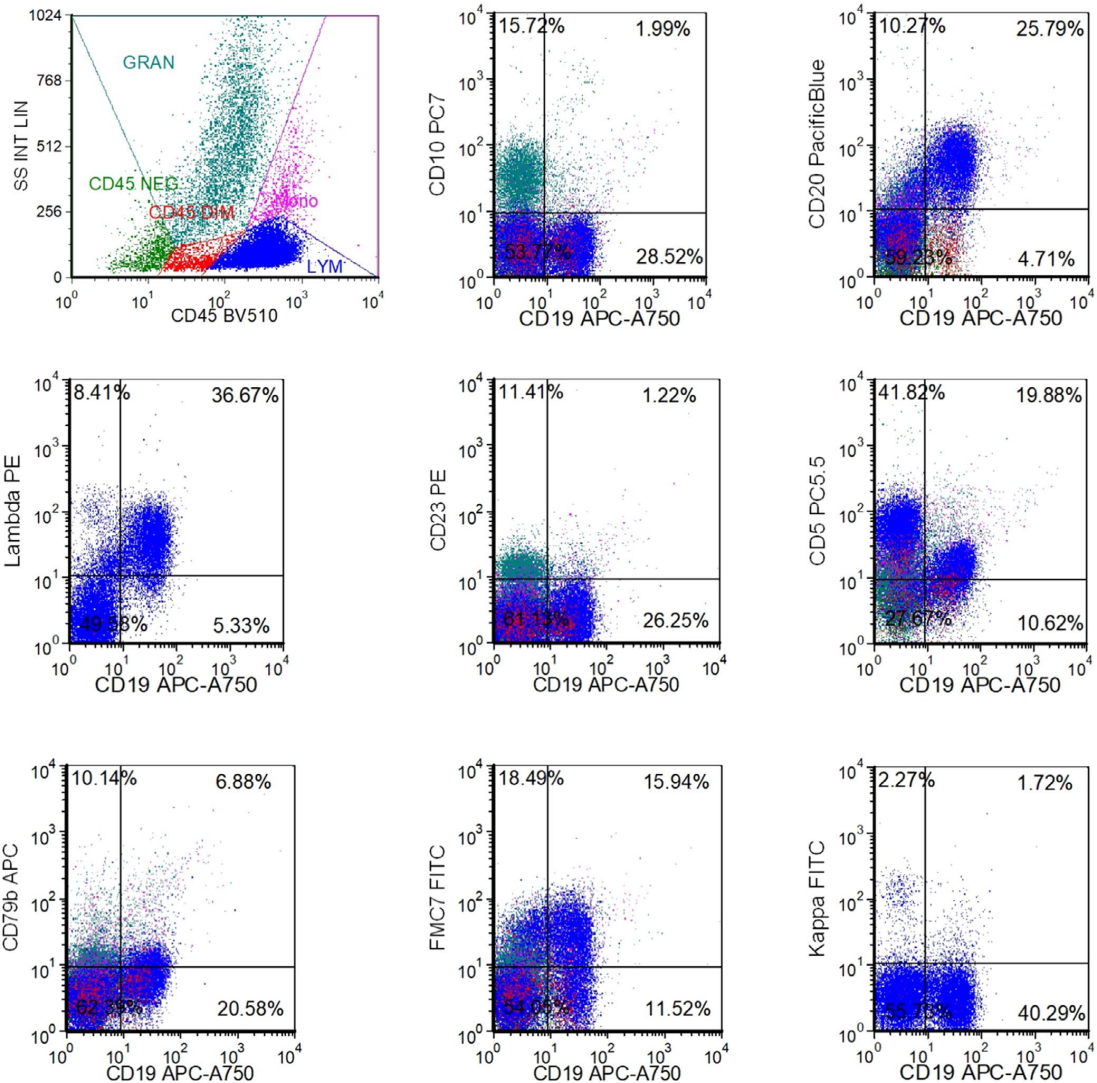
Flow cytometric immunophenotyping confirms a clonal B‐cell population. Analysis of the bone marrow aspirate identified a distinct population of λ‐restricted B‐cells (gated population). The neoplastic cells were positive for CD19, CD20, and showed partial expression of CD5, CD200, and FMC7, with weak CD79b expression. This immunophenotype (Royal Marsden Hospital score = 1) is consistent with a B‐cell lymphoproliferative disorder, confirming the morphological diagnosis.

A bone marrow core biopsy demonstrated hypercellularity (90%) with significant erythroid hypoplasia and an interstitial infiltrate of the atypical lymphocytes (Figure 1E,F). Flow cytometric immunophenotyping analysis conclusively identified a clonal B‐cell population (25.8%) expressing CD19, CD20, and partial CD5, with lambda light chain restriction (Figure 2). The immunophenotype yielded a score of 1 according to the Royal Marsden Hospital system, supporting a diagnosis of a small B‐cell lymphoma.

### 2.4 Differential Diagnosis

The initial differential diagnoses included primary PRCA, primary cold agglutinin disease (CAD), and myelodysplastic syndromes (MDS). The bone marrow erythroid count of 9% effectively ruled out classic PRCA. A diagnosis of MDS was ruled out based on a comprehensive assessment, which included: (1) the absence of definitive morphological features such as increased blasts, trilineage dysplasia, or significant ringed sideroblasts on bone marrow examination; and (2) the absence of supporting genetic evidence, with conventional cytogenetics revealing a normal male karyotype (46,XY[20]) and a targeted next‐generation sequencing panel failing to detect any high‐risk (Type I/II) mutations associated with MDS. The discovery of the clonal B‐cell population by flow cytometry, in conjunction with the morphological findings, ultimately excluded primary CAD and confirmed that the CAD and erythroid hypoplasia were secondary to the underlying B‐cell lymphoma.

### 2.5 Treatment and Clinical Outcome

A diagnosis of small B‐cell lymphoma, not otherwise specified, with associated cold agglutinin disease and secondary PRCA was established. The patient was initiated on a combination therapy of rituximab, ibrutinib, and dexamethasone. The response was remarkable: hemoglobin levels normalized to 132 g/L, the cold agglutinin test became negative, and follow‐up flow cytometry showed no minimal residual disease. A repeat bone marrow examination confirmed recovery of erythropoiesis, with the erythroid series increasing to 26.5%.

## 3 Discussion

We present a highly instructive case whose diagnostic pivot was the observation of erythrophagocytosis and lymphophagocytosis by morphologically atypical, mature lymphocytes—a finding of exceptional rarity that served as the crucial clue to uncovering an underlying B‐cell lymphoma initially obscured by a PRCA‐like presentation and cold agglutinin disease (CAD). This phenomenon transcends a mere morphological curiosity and carries significant implications for diagnostic practice.

The association between acquired PRCA and lymphoproliferative disorders is well‐established, wherein PRCA may precede, coincide with, or follow the diagnosis of lymphoma [5]. The pathogenesis in lymphoma‐associated PRCA is frequently immune‐mediated, often involving autoreactive antibodies produced by the malignant clone that target and inhibit erythroid progenitors [8, 9]. This mechanism is strongly supported in our case by the presence of a high‐titer monoclonal cold agglutinin (IgM) and a positive direct Coombs test, confirming an underlying dysimmune process. The bone marrow erythroid count of 9%, while not fulfilling the strict criteria for PRCA (< 5%), is consistent with a significant, immune‐mediated suppression of erythropoiesis, representing a “PRCA‐like” clinical phenotype driven by the lymphoma.

The central and most striking feature of our case is the phagocytic activity exhibited by the neoplastic B‐cells. Lymphocytes are not professional phagocytes, and such activity is exceedingly uncommon [10]. While isolated instances of platelet or erythrocyte phagocytosis have been documented in conditions like chronic lymphocytic leukemia and T‐cell lymphoma [11, 12, 13], its occurrence remains a remarkable event. The precise mechanism underlying this phenomenon in B‐cell lymphomas is not fully elucidated but may involve aberrant expression of surface receptors or opsonization of blood cells by the paraprotein or autoantibodies (e.g., cold agglutinins) produced by the tumor cells, facilitating recognition and engulfment. Regardless of the exact mechanism, its practical diagnostic value is paramount. The visualization of lymphocyte‐mediated phagocytosis in the context of cytopenias and autoimmune phenomena should be immediately recognized as a high‐value indicator of an underlying lymphoproliferative disorder. It acts as a morphological “smoking gun,” which should directly mandate definitive ancillary studies, such as flow cytometric immunophenotyping, to confirm or exclude lymphoma.

This case offers several critical lessons for clinical practice. First, a diagnosis of PRCA should be approached with caution, and a thorough investigation for an underlying cause, including lymphoma, is mandatory—particularly when associated with immune markers like a positive Coombs test. Second, the macroscopic observation of cold‐induced erythrocyte agglutination in a blood sample is a simple yet powerful clue that should trigger a comprehensive workup for CAD and its underlying etiology, with lymphoma being a prime consideration. Most importantly, our case underscores the indispensable role of meticulous peripheral blood and bone marrow morphology. The identification of the phagocytic lymphocytes was the pivotal step that transformed the diagnostic trajectory. This case provides a generalizable diagnostic lesson: the presence of a distinctive, unexplained morphological clue should actively guide the differential diagnosis and compel a targeted investigation, a principle applicable beyond this specific entity.

A limitation of our report is the inability to definitively characterize the molecular pathways responsible for the phagocytic behavior of the lymphoma cells. Future correlative studies combining detailed immunophenotypic, molecular profiling, and in vitro functional assays could provide deeper insights into this rare biological phenomenon.

In conclusion, this case elevates the observation of phagocytosis by atypical lymphocytes from a rare morphological footnote to a critical diagnostic signpost. We demonstrate that in patients with PRCA‐like manifestations and autoimmune phenomena, this finding is a high‐value indicator of underlying B‐cell lymphoma. Recognizing this signpost should trigger a definitive diagnostic workflow, underscoring how a rare morphological phenomenon can provide a generalizable key to unlocking a complex diagnosis.

## 4 Conclusion

This case highlights that phagocytosis by atypical lymphocytes is a paramount, though rare, diagnostic indicator of underlying B‐cell lymphoma. In patients presenting with erythroid hypoplasia and autoimmune phenomena like cold agglutination, meticulous morphological review for this phenomenon is critical and can guide timely ancillary testing to secure the correct diagnosis.

## Author Contributions

**Tuo Cao:** conceptualization, data curation, investigation, writing – original draft. **Yuting Li:** conceptualization, methodology. **Xiangming Peng:** data curation, funding acquisition, writing – review and editing.

## Funding

This work was supported by the Joint Municipal‐University (Hospital)‐Enterprise Funding Program of the Guangzhou Science and Technology Bureau, Grant No. 2023A03J0988.

## Consent

Written informed consent was obtained from the patient for the publication of this case report and any accompanying images.

## Conflicts of Interest

The authors declare no conflicts of interest.

## Data Availability

The data that support the findings of this study are not publicly available due to privacy and ethical restrictions, as they contain sensitive patient information.
